# Prevalence and risk factors of hypertension among public servants in Ejisu-Juaben municipality, Ghana

**DOI:** 10.1186/s13104-023-06349-4

**Published:** 2023-05-15

**Authors:** Bernice Agyemang-Pambour, Isaac Osei, Estella Antoinette Boateng-Osei, Alexander Kwarteng, Veronica Dzomeku

**Affiliations:** 1 Department of Nursing, Faculty of Allied Health Sciences, Kwame Nkrumah University of Science and Technology, Kumasi, Ghana; 2 Medical Research Council Unit The Gambia at London School of Hygiene & Tropical Medicine, PO Box 273, Banjul, West Africa, The Gambia; 3 Faculty of Infectious and Tropical Diseases, London School of Hygiene & Tropical Medicine, London, UK; 4 Department of Biochemistry and Biotechnology, Kwame Nkrumah University of Science and Technology, Kumasi, Ghana

**Keywords:** Hypertension, Blood pressure, Public servants, Ghana, Risk factors

## Abstract

**Objectives:**

We determined the prevalence and risk factors of hypertension among public servants of Ejisu Juaben municipality.

**Results:**

The overall prevalence of hypertension was 29.3% (95%CI:22.5–36.1%) and only 8.6% of the participants were aware of their hypertensive status. Respondents who were > 40 years were twice as likely to develop hypertension compared to those who were ≤ 40 years [adjusted odds ratio (AOR) = 2.37, 95% confidence interval (CI) 1.05–5.32]. Those who were married were 2.54 times more likely to be hypertensive compared with those unmarried [AOR = 2.54, 95%CI: 1.06–6.08]. Compared to health workers, Judicial and Security service workers were almost five times more likely to be hypertensive [AOR = 4.77, 95%CI: 1.20–18.96]. Being overweight [AOR = 2.25, 95%CI: 1.06–6.41] and obese [AOR = 4.80, 95%CI: 1.82–12.91] was associated with increased odds of hypertension. The prevalence of hypertension among the participants in this study is high. Employee wellness programs are needed at workplaces and the Ghana Health Service must adopt targeted intervention programs such as regular screening for non-communicable diseases and promotion of physical activities at the workplace.

## Introduction

Hypertension is one of the primary causes of premature death worldwide. It accounted for about 10.4 million deaths worldwide in 2017 [1]. Globally, an estimated 1.13 billion people are known to have hypertension, which is projected to affect 20% of the world population by 2025 [1, 2]. It is the most significant risk factor for cardiovascular-related deaths and morbidity worldwide. Most (70%) of the affected populations reside in lower-middle-income countries (LMICs)[3].

In people less than 60 years of age, while hypertension accounts for 7% of mortality in developed countries, in Sub-Saharan Africa, it is responsible for 25% of deaths [4]. The prevalence of hypertension in sub-Saharan Africa has been increasing over the past decades. Findings from a systematic review among adults in sub-Saharan Africa showed a pooled prevalence of 57.0% ranging from 22.3 to 90.0% [5]. Over the past decades, there has been a paradigm shift in disease burden from communicable to non-communicable in most developing countries. Sub-Saharan Africa is now confronted with a double burden of both communicable and non-communicable diseases [6]. The rapid increase in non-communicable diseases in most African countries including Ghana has been attributed to globalization, rapid urbanization, and unhealthy lifestyles such as unhealthy diets, lack of physical activity, alcohol consumption, and tobacco use [7].

The 2014 Ghana Demographic and Health Survey indicated that 13.1% of adults aged 15–49 years had hypertension [8]. Due to sedentary work that requires sitting for long hours, heavy workload demands, lack of support at work, and other work-related stress factors, public servants are a plausible high-risk group for developing hypertension [9, 10]. Although several hypertension studies have been conducted in Ghana, most have been conducted among the general population [11–13] whiles the few which specifically targeted public servants were conducted over a decade ago [14, 15]. This study aimed to determine the prevalence and risk factors of hypertension among public servants of Ejisu-Juaben Municipality, Ghana.

## Materials and methods

### Study setting

**Fig. 1:**
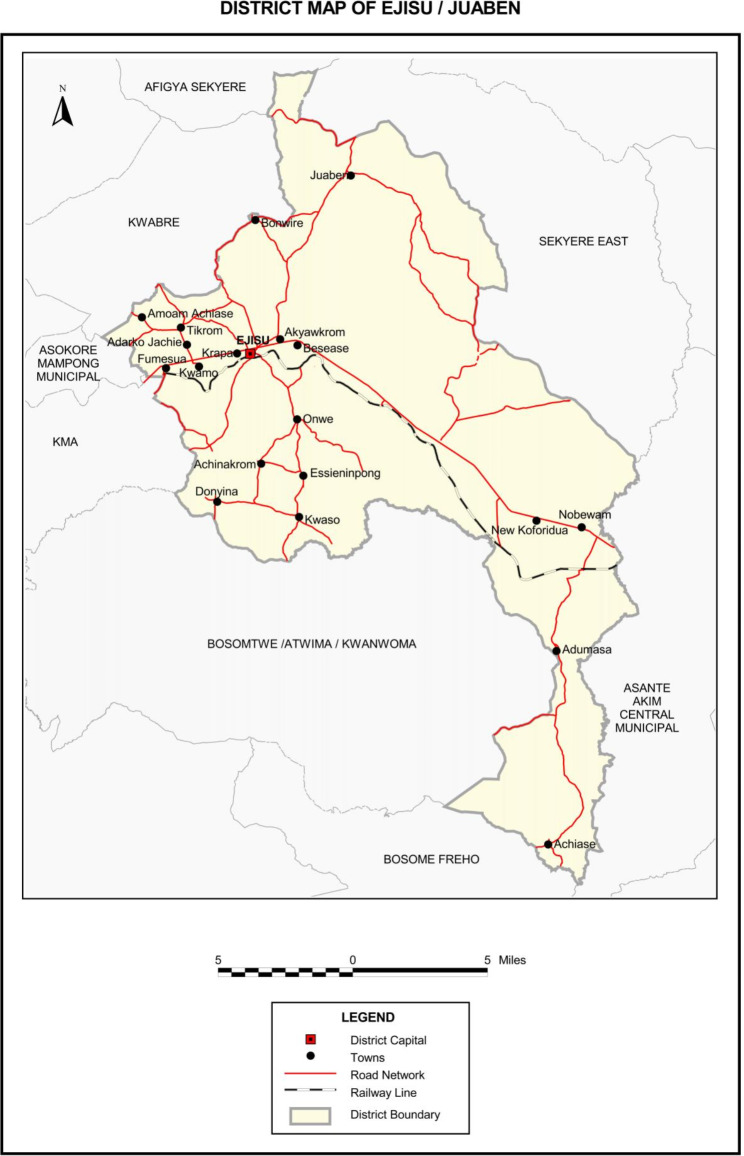
Map of Ejisu-Juaben Municipality. Source: Ghana Statistical Service, [16]

The study was conducted in the Ejisu-Juaben Municipality in the Ashanti Region. The Municipality is among the 30 administrative and political districts in the Ashanti Region of Ghana. The Municipality is positioned within the central part of the Ashanti Region and shares borders with six other districts in the region with Ejisu as its capital. The Municipality was selected due to its many public service departments compared to surrounding districts (Fig. 1).

### Study design

A cross-sectional study was conducted among public servants in the Ejisu-Juaben Municipality. A public servant in this study refers to an individual whom the state or any government establishment has contracted by Sect. 4 of the Civil Service Act: 1993 (PNDCL 327) of Ghana and who is on the government of Ghana payroll within the Ejisu-Juaben Municipality [17].

### Sample size

A recent study estimated a national hypertension prevalence of 13.1% [8]. Based on the study area’s estimated public servant population size of 3200, considering a confidence level of 95% with a precision of 0.05, a sample size of 165 was derived. Consequently, to adjust for non-responses, a final sample size of 174 participants was determined. We used the software provided by Select Statistical Services (https://select-statistics.co.uk/) to calculate the sample size.

### Inclusion and exclusion criteria

Participants who were on the government payroll and in active service within the Ejisu Juaben Municipality were included in the study. Public Servants who were pregnant at the time of the survey and those who did not consent to participate in the study were excluded.

### Sampling method

The list of total employees of 8 public service departments in the Municipality namely: the Local Government, Forestry Commission, Judicial Service, Police Service, Fire Service, Immigration Service, Education, and Health Services were obtained from the Ejisu-Juaben Municipal Assembly. Using probability proportionate by size, the number of respondents selected from each of the departments was estimated. We used convenience sampling to select the participants.

### Data collection technique and tool

We used a modified WHO STEPS instrument and global physical activity questionnaire (GPAQ) to collect data from the respondents [18, 19]. The questionnaire was piloted on a small number of selected public servants in the study area. The modified tool was structured into three parts: socio-demographic characteristics; lifestyle habits and physical activity; and anthropometric and blood pressure measurements. Data were collected from July 18, 2018, to August 15, 2018.

### Operational definitions

#### Body Mass Index (BMI)

We classified BMI using the World Health Organization standard definitions: underweight was classified as a BMI < 18.5 kg/m^2^, normal weight 18.5–24.9 kg/m^2^, overweight 25.0–29.9 kg/m^2^, and obese ≥ 30.0 kg/m^2^ [20]. We calculated the BMI as weight in kilograms divided by height in meters squared.

#### Hypertension

We defined hypertension based on the classification by the 7th Joint National Committee on Prevention, Detection, Evaluation, and Treatment of High Blood Pressure’s report of 2003. A mean systolic blood pressure of ≥ 140 mmHg systolic and/or ≥ 90 mmHg diastolic was considered hypertensive [21].

### Physical measurements

#### Blood pressure

Blood pressure (BP) measurements were performed on the left arm of respondents in a sitting position using the Omron digital BP monitor (Omron Healthcare Co. Kyoto, Japan) with a suitable adult cuff. Two BP measurements were taken on each respondent at 5 min intervals and the mean of the two measurements was assigned as the final BP of the respondents.

#### Body weight and height

Respondents’ weight and height were measured using a standard stadiometer fixed to a calibrated weighing scale. Weights and heights were measured with respondents in an upright position, back and heels against the stadiometer, facing forward with hands hanging loosely by the sides, and wearing light clothes with no footwear. Weight and height readings were expressed in kilograms (kg) and to the nearest centimeter (cm), respectively. These procedures were carried out by the manufacturer’s instructions.

### Statistical analysis

Data were entered into Microsoft Excel and exported to STATA version 17 (STATA Corp., Texas, USA) for analyses. A summary of the data was examined using descriptive statistics involving frequencies and percentages. Binary logistic regression was performed to identify risk factors for hypertension. Variables with p values < 0.2 were entered into a multivariable logistic regression model to determine the risk factors associated with hypertension adjusting for other covariates in the model. Normality and multi-collinearity assumptions were assessed. We presented both crude and adjusted odds ratios and determined a statistical significance at a 95% confidence interval and a *p*-value of < 0.05.

## Results

A total of 174 participants from eight public service departments were enrolled. The mean (+/-SD) age of the participants was 34.7(+/-7.6) years, with 75.2% below 40 years of age. There were more females (57.5%), 88% of the participants were Christians and 61.5% were married. The majority (82.2%) had completed tertiary education and 49.4% worked in the Education service. Fifteen (8.6%) participants had previously been diagnosed as hypertensive by a health worker. There were no current cigarette smokers and 13.5% reported consuming alcohol in the last 7 days before the survey. Twenty-eight (16.5%) of the participants were not involved in any physical activities (Table 1).

**Table 1:** Sociodemographic characteristics and lifestyle factors of study participants

Characteristics	Categories	Number (n)*	Percent (%)
Age, years (n = 153)	Younger (≤ 40)	115	75.2
	Older (> 40)	38	24.8
Sex (n = 174)	Male	74	42.5
	Female	100	57.5
Religion (n = 173)	Christians	153	88.4
	Muslim	20	11.6
Marital Status (n = 174)	Married	107	61.5
	Unmarried	67	38.5
Highest Education Level (n = 174)	Pre-Tertiary	31	17.8
	Tertiary	143	82.2
Previously diagnosed as hypertensive (n = 174)	Yes	15	8.6
	No	159	91.4
Occupation (n = 174)	Local government^†^	16	9.2
	Judicial and Security services^λ^	16	9.2
	Health workers	56	32.2
	Education service	86	49.4
Current cigarette smoker (n = 170)	Yes	0	0
	No	170	100
Alcohol consumption (n = 170)	Yes	23	13.5
	No	147	86.5
Vigorous physical Activities at least 30 min (No of days per week) (n = 170)	None	28	16.5
	1 – 2	63	37.0
	3 – 4	41	24.1
	≥ 5	38	22.4
BMI^**^ (kg/m^2^) (n = 173)	Normal weight	78	45.1
	Overweight	58	33.5
	Obesity	37	21.4

^*^Due to missing values not all n values equal 174 i.e., Age (21), Religion (1), Alcohol consumption (4), smoking (4), physical exercise (4), BMI (1)

**BMI was redefined into 3 categories due to low numbers of underweights (normal weight < 25.0 kg/m^2^, overweight 25.0–29.9 kg/m^2^, and obese ≥ 30.0 kg/m^2^)

†Local government includes workers from Forestry Commission

λ security services include Police, Fire service, Immigration

### Physical measurements

The prevalence of hypertension among the respondents was 29.3% (95%CI:22.5–36.1%). The mean (+/-SD) systolic blood pressure was 124.83 (± 16.68) mmHg, and the mean (+/-SD) diastolic blood pressure was 81.55 (± 14.63) mmHg. The mean (+/-SD) BMI of the study respondents was 26.4 (+/- 5.1). A third (33.5%) and a fifth (21.4%) of the participants were classified as overweight and obese respectively (Table 1).

### Factors associated with hypertension

In the bivariate analysis, age, sex, marital status, occupation, vigorous physical exercise, and BMI were found to have p-value < 0.2 and were included in the multivariable logistic model. In the final multivariable logistic model, age, marital status, type of occupation, and BMI remained significantly associated with hypertension. Respondents who were > 40 years were twice as likely to develop hypertension compared to those who were ≤ 40 years [AOR = 2.37, 95%CI:1.05–5.32]. The odds of hypertension were 2.54 times higher in those who were married compared to unmarried participants [AOR = 2.54, 95%CI: 1.06–6.08]. Compared to health workers, the odds of hypertension were 4.77 times higher among Judicial and Security service participants [AOR = 4.77, 95%CI: 1.20–18.96]. Being overweight [AOR = 2.25, 95%CI: 1.06–6.41] and obese [AOR = 4.80, 95%CI: 1.82–12.91] was associated with increased odds of hypertension (Table 2).

**Table 2:** Bivariate and multivariable logistic regression analysis of factors associated with hypertension among public servants at Ejisu-Juaben Municipality

Characteristics	Hypertension		Bivariate COR (95% CI)	Multivariable AOR (95% CI)	P-value
	Yes	No			
Age, years					
≤ 40	28	87	1	1	1
> 40	17	21	2.51 (1.17–5.42)	2.37 (1.05–5.32)	0.04
Sex					
Male	26	48	1.63 (0.84–3.14)	1.57 (0.70–3.52)	0.27
Female	25	75	1	1	1
Marital Status					
Married	40	67	3.04 (1.43–6.47)	2.54 (1.06–6.08)	0.04
Unmarried	11	56	1	1	1
Occupation					
Health workers	8	48	1	1	1
Local government	4	12	2 (0.52–7.77)	1.64 (0.35–7.61)	0.53
Judicial and Security services	9	7	7.71 (2.23–26.64)	4.77 (1.20–18.96)	0.03
Education service	30	56	3.21 (1.35–7.67)	2.55 (1.00–6.40)	0.05
Vigorous physical Activities at least 30 min (No of days per week)					
None	8	20	1	1	1
1 – 2	25	38	1.64 (0.63–4.31)	1.06 (0.35–3.22)	0.92
3 – 4	9	32	0.70 (0.23–2.12)	0.80 (0.24–2.74)	0.73
≥ 5	7	31	0.56 (0.18–1.80)	0.45 (0.12–1.73)	0.24
BMI (kg/m^2^)					
Normal weight	12	66	1	1	1
Overweight	20	38	2.89 (1.28–6.57)	2.60 (1.06–6.41)	0.04
Obesity	18	19	5.21 (2.14–12.70)	4.80 (1.82–12.91)	0.002

COR = Crude Odds Ratio, AOR = Adjusted Odds Ratio

## Discussion

The overall prevalence of hypertension was 29.3% among respondents. Age, marital status, occupation, and BMI were risk factors found to be significantly associated with hypertension. The prevalence of hypertension is consistent with similar studies conducted among Public servants in Addis Ababa (27.3%) [22], Nigeria (27.8%) [23], and workers in Kenya (30.1%) [24]. However, our finding is higher than comparative studies conducted among public servants in Ghana (20%) [25], Northern Ethiopia (16%) [26], and Southern Ethiopia (24.5%) [27]. The reason for the differences in hypertension prevalence may be due to the setting and other sociodemographic factors such as age differences among the study participants. The studies with higher hypertension prevalence, including this study were conducted mainly among urban dwellers and most of the participants were 30 years and above, while those with low prevalence were mainly conducted in rural areas and participants 18 years and above. Urbanization has been recognized as a major driving force for the increase in chronic conditions such as hypertension [28].

In this study, increased age was significantly associated with hypertension. This finding is comparable to published studies [11, 22, 26]. Increasing age has been established to be associated with hypertension. A study among federal ministry civil servants in Addis Ababa, Ethiopia, showed that civil servants who were 48 years and above were six times more likely to be hypertensive compared to those aged 18–27 years [26]. The stiffening of the arterial wall due to structural physiological changes associated with aging has been attributed to an increased risk of hypertension with age [29].

We found that being married was significantly associated with hypertension. Similar studies conducted in Ethiopia [30] and Iran [31] have reported a higher prevalence among married participants. However, other studies have also shown that being married is protective against hypertension [32, 33]. Compared to unmarried, married couples are prone to marriage-related stress conditions such as child-rearing, bills, and mortgages and these may explain the findings in our study.

Judicial and Security service workers were found to be almost five times more likely to be hypertensive as compared to healthcare workers. This finding is consistent with published literature. A recent study conducted in Israel found that healthcare workers adopted better healthy lifestyles in nutrition, physical activity, and health responsibility than workers in other professions [34]. A similar finding was reported in a study in North America where healthcare professionals as compared to the general population reported better health behaviours in smoking and physical activity [35]. Healthcare workers are expected to be more knowledgeable than the general population concerning healthcare behaviors and consequences. Additionally, most healthcare workers might perceive themselves as role models for their patients and the general population and this encourages them to adopt a healthier lifestyle, which may explain the finding in this study.

Participants in this study who were classified as overweight and obese had higher increased odds of hypertension compared to those with normal BMI. This finding is consistent with reports from previous studies conducted among workers in Ghana [25, 36]. Our study showed that only about a fifth of the participants adhered to WHO recommendations on physical activity for health, i.e., respondents engaging in at least 30 min of physical exercise 5 or more days a week. The lack of exercise and sedentary lifestyle could explain the high blood pressure among participants classified as overweight and obese.

## Conclusion

The prevalence of hypertension among the participants in this study is high. This study showed that age, marital status, occupation, and BMI were the risk factors for hypertension among public servants. Public servants are one of the main driving workforces of the country [37], and this finding presents a public health concern. Employee wellness programs are needed at workplaces and the Ghana Health Service must adopt targeted intervention programs such as regular screening for non-communicable diseases and promotion of physical activities at the workplace.

### Limitation

This study has some limitations. The sample size was small, a history of anti-hypertensive was not collected, there were missing data on age, and convenience sampling was used which might introduce bias. This is a cross-sectional study, and the findings should be interpreted with caution as causal inference and temporality cannot be established.

## Data Availability

Data will be made available by the corresponding author upon reasonable request.

## Abbreviations

LMIC: Lower-middle-income countries
BMI: Body Mass Index
BP: Blood Pressure
COR: Crude Odds Ratio
AOR: Adjusted Odds Ratio
GPAQ: Global physical activity questionnaire
STEPS: STEPwise approach to NCD risk factor surveillance
WHO: World Health Organization
PNDCL: Provisional National Defense Council Law
